# Oral health, dental treatment, and medication related osteonecrosis of the jaw in multiple myeloma – a longitudinal cohort study

**DOI:** 10.1186/s12903-024-03943-1

**Published:** 2024-02-05

**Authors:** Richard Olofsson, Magdalena Korytowska, Ulrica Almhöjd, Annica Almståhl, Hülya Cevik-Aras

**Affiliations:** 1 Specialist Clinic for Orofacial Medicine Uddevalla - Trollhättan, Public Dental Service, Region Västra Götaland, Uddevalla, Sweden; 2 Department of Oral Microbiology and Immunology, Institute of Odontology, Sahlgrenska Academy, University of Gothenburg, Göteborg, Sweden; 3 Specialist Clinic for Orofacial Medicine, Karlstad, Public Dental Service, Region Värmland, Sweden; 4 Department of Oral Maxillofacial Surgery and Oral Medicine, Malmö University, Malmö, Sweden; 5 Department of Cariology, Institute of Odontology, Sahlgrenska Academy, University of Gothenburg, Gothenburg, Sweden; 6 Section 4 – Oral health, Faculty of Odontology, Malmö University, Malmö, Sweden; 7 Department of Oral Pathology and Medicine, Institute of Odontology, Sahlgrenska Academy, University of Gothenburg, Gothenburg, Sweden

**Keywords:** Bisphosphonates, Dental surgery, Medication related osteonecrosis of the jaw, Multiple myeloma, Oral health, Oral health related quality of life

## Abstract

**Objective:**

The objective of the present study was to investigate oral health status, oral health related quality of life, and identify risk factors associated with invasive dental treatment and medication related osteonecrosis of the jaw in patients with multiple myeloma.

**Material and methods:**

Patients newly diagnosed with multiple myeloma (*n* = 144) referred between January 2015 and September 2022 were retrospectively included. The patients underwent a thorough clinical and radiological oral examination and odontogenic infections were treated before the start of bisphosphonate treatment. The patients were followed annually, including clinical and radiological examinations. The oral health related quality of life was investigated by the OHIP-14 questionnaire.

**Results:**

Dental treatment (RR = 7.75), receiving combination antineoplastic therapy≥3 (RR =4.13), periodontitis (RR = 4.21), and reduced number of teeth (RR = 2.87) were associated with an increased risk of medication related osteonecrosis of the jaw. The response rate of the OHIP-14 questionnaire was 70.2%. Oral pain or discomfort in the mouth related to the medical treatment was reported by 30.5%.

**Conclusion:**

Dental screening and treatment planning in patients with Multiple Myeloma may result in fewer oral infections and fewer interruptions of the medical treatment of myeloma.

## Introduction

Multiple myeloma (MM) is a malignant haematological disease originating from the lymphatic B cell system in the bone marrow. The global incidence of myeloma is 160,000 cases/year, with a mortality rate of 106,000 people/year. Mean survival in MM patients < 65 years is 8.7 years and among patients > 65 years 3.8 years [1]. The aetiology is still unknown, but increased age and monoclonal gammopathy are considered to increase the risk of developing myeloma [2]. The malignant plasma cells infiltrate the bone tissue, producing osteolytic cytokines, which further leads to general osteopenia or osteolytic bone lesions. Also, the malignant cells produce monoclonal antibodies and free light chains that may deposit and cause renal impairment [3]. During intense disease burden patients may develop pancytopenia, hypercalcaemia, renal failure, and pathological bone fractures [4, 5]. A vast range of treatment modalities exists, such as antiresorptive treatments, chemotherapy, immunomodulatory agents, proteasome inhibitors and corticosteroids [6]. Antiresorptive agents are the most frequently used treatment alternative in MM to reduce osteolytic activity and hypercalcaemia. The most common antiresorptive agents in myeloma treatment are zoledronic and pamidronic acids, highly potent bisphosphonates (BP), which are given every 4 weeks during disease burden. Antiresorptive agents produce a severe adverse side effect as it increases the risk of Medication-Related Osteonecrosis of the Jaw (MRONJ). MRONJ is defined as exposed jawbone or bone that can be probed through a fistula persisting > 8 weeks in a patient who has been treated with antiresorptive agents alone or in combination with immune modulators or antiangiogenic medications with no history of radiation therapy to the jaws. The condition can be very painful and lead to reduced nutrition, severe bone infections, and pathological fracture of the mandible [7]. Besides, during periods of pancytopenia, painful oral ulcers or haematomas may appear, caused by fractured teeth or ill-fitting dentures [8]. In MM, invasive dental treatment during pancytopenia was shown to increase the risk of bleeding complications and bacteraemia, whilst BP or irradiated jaw increase the risk of osteonecrosis [9].

One previous study mentioned the importance of supportive dental care programs (dental examination, treatment of periapical lesions by endodontic treatment or, if the prognosis is poor, surgical removal of teeth and optimisation of oral hygiene) in MM patients before the start of BP treatment to reduce the risk of MRONJ adverse effects of antineoplastic treatment [10]. For patients treated with high-dose BPs, the risk of developing MRONJ has been considered life-long since the half-life of BP in bone is very long, around 12 years [11].

At diagnosis of MM, 80% of the patients present with osteolytic lesions in the bone tissue, of which 20–30% manifest in the mandible [12]. Osteolytic bone lesions may cause pain, tooth mobility, loss of sensation and, in the worst cases, pathological fractures of the mandible or oroantral communication of the maxilla [13, 14]. Regarding the odontological aspects of MM, there are only a handful of studies. Feitosa et al. investigated the oral health in a small cohort [15], further the management of oral mucositis during HSCT (haematopoietic stem cell transplantation), and few case reports describe soft tissue/jaw manifestation of MM lesions has been described [16–18]. Oral Health-Related Quality of Life (OHRQoL) in myeloma has been investigated during HSCT, indicating impaired OHRQoL with the greatest impact on functional limitation, physical pain, and physical disability; however, no previous study has investigated OHRQoL in a cross-section of patients with MM [19].

To our knowledge, none of the previous studies has focused longitudinally on oral health status and risk factors associated with dental intervention and MRONJ development in patients with multiple myeloma.

Accordingly, the primary aim of this study was to investigate oral health status, Oral Health-Related Quality of Life and risk factors associated with MRONJ due to dental treatment after BP. Moreover, the incidence of osteolytic lesions in the mandible and maxilla in MM was studied.

## Methods

The study was performed according to the STROBE checklist for cohort studies [20] and all study protocols approved by the Swedish Ethical Review Authority with registration number 2020–04620.

**Fig. 1:**
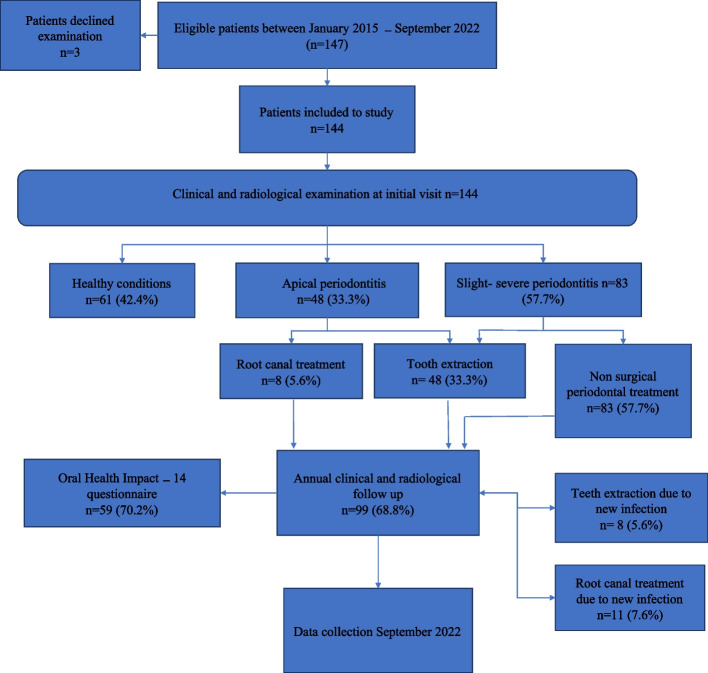
Scheme of the study design

All patients with newly diagnosed MM, referred to the Specialist Clinic for Orofacial medicine in Uddevalla and Trollhättan, Sweden, between January 2015 and August 2022, were included according to the local routines for patients with newly diagnosed MM (Fig. 1). Patients who did not undergo the clinical and radiological examinations or declined follow-up visits were excluded from the study (*n* = 3). The patients underwent a thorough clinical and radiological dental examination including intraoral bitewing, periapical and panoramic x-rays. Data regarding past and present diseases and medication had been collected through medical journals. Data regarding tobacco use and oral hygiene habits had been collected by asking the patient. The clinical examination consisted of inspection and palpation of the oral tissues, recording of present teeth, filled teeth, and decayed teeth, and periodontal status by recording of probing pocket depth, bleeding on probing, clinical attachment loss (CAL), furcation involvement, and tooth mobility. Periodontal health was classified as healthy, slight periodontitis (CAL 1–2 mm), moderate periodontitis (CAL 3–4 mm), and severe periodontitis (CAL > 5 mm) according to the definition developed by the working group of the Centre of Disease Control and Prevention in collaboration with the American Academy of Periodontology [21]. The intraoral dental X-rays were evaluated by the dental practitioners and the panoramic X-rays by oral and maxillofacial radiologists to diagnose the presence of any dental infections and/or osteolytic lesion in the jaws. If present, infections were treated by extraction or root canal treatment. In the patients who received tooth extractions, healing was checked to ensure good primary healing with full mucosal coverage and no probeable bone prior the start of BP. In case of root canal treatment instrumentation was done prior to the initiation of BP. The patient received oral and written information about the importance of maintaining good oral hygiene, potential oral adverse effects of the medical treatment and the risk of MRONJ development due to BP. Appropriate fluoride prophylaxis and oral hygiene routines were recommended based on individual assessments. The patient was then seen annually for clinical and radiological follow-up, the radiological follow up consisted of panoramic X-rays and in case of uncertainty regarding apical status it was supplemented with intraoral periapical X-rays. In case of MRONJ development either clinically or radiologically diagnosis and staging were performed according to the AAOMS guidelines, the responsible haematologist was informed of the condition and the patients received non operative treatment and/or operative treatment depending on severity, symptoms, and general condition of the patient [7]. If signs of periodontal disease, bleeding on probing and periodontal pocket probing depth > 5 mm were present, treated by a dental hygienist, including instrumentation, information, and instruction in oral hygiene. New onset apical periodontitis discovered during annual check-ups or due to emergency visits was treated with root canal treatment by specialist in endodontics under antibiotic prophylaxis with Amoxicillin 2 g one-hour prior treatment or Clindamycin 300 mg in case of penicillin allergy. Teeth with aggravated periodontitis, or apical periodontitis that has been unable or failed root canal treatment were extracted surgically with mucoperiosteal flap and resection of sharp bone edges followed by primary closure of the wound. These patients received antibiotic treatment with Phenoxymethylpenicillin 1.6 g × 3 for 7 days or Clindamycin 150 mg × 3 for 7 days, and healing control within 3 weeks.

The annual follow-ups continued until September 2022 or until the death of the patient.

## Clinical and radiological data

All data collection was carried out retrospectively during September 2022. Dental and medical records were used to collect the data as mentioned below.

Medical status:

- Age and gender.
- Comorbidity and survival time.
- Anti-neoplastic treatment.

Dental status (pre and post BP):

- Number of teeth, DMFT (Decayed, Missed and Filled Teeth) [22].
- Periodontal status: grouped into healthy/mild periodontitis and moderate/severe periodontitis [21, 22].
- Periapical status according to the WHO oral health surveys [22].
- Tooth extractions and endodontic treatments performed pre and post BP.
- Diagnosis of MRONJ according to AAOMS guidelines [7].
- Radiological findings; presence of OL lesions in jaw bones.

Comorbidity was determined according to the validated Adult Comorbidity Evaluation 27 (ACE-27) instrument by using the Cancer Comorbidity Calculator. The ACE-27 score ranges from 0 = no comorbidity, 1 = mild, 2 = moderate, and 3 = severe [23]. Survival time was measured in months from diagnosis to death or the date of data collection. Time until invasive dental treatment (extractions and root canal treatment) and MRONJ was measured in months from the first dental examination until the recording of an event in the dental records.

## Oral health-related quality of life

To investigate the Oral Health-Related Quality of Life (OHRQoL) a prospective part was added to the study using the Oral Health Impact 14 (OHIP-14) questionnaire in Swedish, which has shown good validity and reliability [24, 25]. It consists of 14 questions distributed over seven domains: functional limitation, physical pain, physical discomfort, physical disability, psychological disability, social disability, and handicap. The response options were 0–4; never to very often. Three additional questions especially developed for the study were also asked:

- Have you experienced pain or discomfort in the mouth related to the medical treatment?
- Have you received any treatment due to pain or discomfort in the oral cavity?
- Have you experienced that you cannot perform oral hygiene because of your general health?

Among the previously described patients, those who were diagnosed with MM in 2018 or later (*n* = 83) were asked by letter to participate in the OHRQoL part of this study. The survey was sent out every December to those diagnosed with MM in the last 15 months and at least 3 months must have passed since their first dental examination. The letter included written information about the study, a written consent form, the questionnaire, and a stamped return envelope. The questionnaire was coded and separated from the consent form by an administrator on arrival. Patients who were deceased or unable to give informed consent were excluded from this part of the study. Participants were not reminded to answer the survey. To analyse the physical, psychological, social, psychosocial, and pain-discomfort impacts on OHRQoL, the questions were grouped into first, second and third order factors, as described by Campos et al. [26].

## Statistical analysis

Microsoft® Excel® for Microsoft 365 MSO (Version 2208 Build 16.0.15601.20526) software was used for the analysis of descriptive data and OHIP-14. The OHIP-14 data were described by mean value.

The data were divided into categorical and numerical values. Gender, periodontal stage, ACE-27 classification, antineoplastic treatment, dental treatment pre/post BP and MRONJ were defined as categorical variables. The numerical variables consisted of age, follow-up time, decayed teeth, missed teeth, periapical lesions, months till dental treatment after the first dental examination and months till MRONJ after the first dental examination.

Invasive dental treatment and MRONJ were described by incidence, incidence rate and attributable fraction. Osteolytic bone lesions were described by prevalence and relative risk of death.

To examine the association between numerical and categorical variables at inclusion and invasive treatment/MRONJ, the relative risk with 95% confidence interval was calculated. To analyse confounding factors, the measure of association between the variables was investigated by the distribution of prevalence.

## Results

### Clinical and demographical data

A total of 147 patients were diagnosed with MM and referred between January 2015 and September 2022. Three patients had declined the first oral examination due to impaired conditions and were excluded from the study. The remaining 144 were included in the study.

The majority of the patients were male 66.7% (*n* = 96) and the mean age was 73.5 ± 9.8 years. The median total follow-up time of the patients was 22.5 months.

The median ACE score was 1.5 and the most common comorbidities were hypertension (*n* = 86, 59.7%), hyperlipidaemia (*n* = 47, 32.5%), and atrial fibrillation (*n* = 28, 19.4%). Immunomodulatory drugs in combination with corticosteroids and BP were the most common anti-neoplastic medications received by the patients. A triplet regimen was given to 46.5% of the patients (*n* = 67), while 45.1% (*n* = 65) received double and 8.3% (*n* = 12) single treatment. In all patients the BP treatment consisted of a 4 mg Zoledronic acid infusion every 4 weeks for a duration of 2 years and the terminated. The treatment was resumed upon disease progression with Zoledronic acid infusion every 4–12 weeks. BP was received by 95% of the patients (*n* = 137), 4.2% (*n* = 6) died before BP treatment could be initiated, and 0.7% (*n* = 1) did not need treatment.

At the initial visit, 26.8% of the patients (*n* = 39) were diagnosed with manifest caries lesions, 33.3% (*n* = 48) with periapical lesions, 18.1% (*n* = 26) with moderate periodontitis, and 6.3% (*n* = 9) with severe periodontitis. Teeth with extensive caries/periapical lesions and/or severe periodontal disease were either extracted (*n* = 48, 33.3%) or treated with root canal treatment (*n* = 8, 5.6%) to eliminate odontogenic infections prior to BP treatment. Manifest caries lesions were restored if treatable (*n* = 13, 9%). Periodontal treatment was given to 57.7% of the patients (*n* = 83) by a dental hygienist. The clinical and demographic characteristics of the patients are further described in Table 1.

**Table 1:** Clinical and demographic characteristics of the myeloma patients at the first dental examination (*n* = 144)

**Age, year median (range)**	76 (49–93)
**Gender n (%)**	
Male	96 (66.7)
Female	48 (33.3)
**Comorbidity n (%)**	
**Adult comorbidity evaluation −27, median (range)**	1.5 (0–3)
3	18 (12.5)
2	55 (38.2)
1	55 (38.2)
0	16 (11.1)
**Number of antineoplastic treatments, median (range)**	2 (0–7)
**Patients undergoing HSCT**^**1**^ **n (%)**	28 (19.4)
**Follow-up time, months (range)**	22.5 (1–92)
**Dental status**	
Number of teeth, median (range)	24 (**0**–28)
Edentulous patients n (%)	4 (2.8)
DMFT^2^, median (range)	21 (4–28)
Decayed teeth, median (range)	0 (0–9)
Missed teeth, median (range)	4 (0–28)
Filled teeth, median (range)	14 (0–23)
**Periodontal status n (% of patients)**	
No periodontitis	61 (42.3)
Slight periodontitis	48 (33.3)
Moderate periodontitis	26 (18.1)
Severe periodontitis	9 (6.3)
**Periapical status n (% of patients)**	
Periapical lesion (%)	48 (33.3)
**Applied treatment prior to BP**^**3**^ **n (% of patients)**	
Tooth extraction n (%)	48 (33.3)
Root canal treatment n (%)	8 (5.6)
**Removable prosthetics n (% of patients)**	22 (15.3)

Data are presented as numbers and percentages or numbers, median and range

1. *HSCT* Haematopoietic stem cell transplantation

2. *DMFT* Decayed, missed and filled teeth

3. *BP* Bisphosphonate treatment

### Invasive dental treatment

After the start of BP treatment, 17 patients (11.8%) received invasive dental treatment (tooth extraction and/or root canal treatment, due to teeth diagnosed with periapical lesions or aggravated periodontal disease, at the follow-up examinations. Of these 17 patients, 78.8% (*n* = 13) were males and 22.2% (*n* = 4) were females with a median age of 73 years (range 56–85 years). The mean time until invasive dental treatment after the first dental examination was 23.4 ± 19.7 months. The incidence rate of invasive dental treatment was 7.7/100 person-years. The median ACE-27 comorbidity score among these patients was slightly higher [2] than the average score (1.5). Seventeen patients received invasive dental treatment, involving a total of 27 teeth. Out of these, 13 teeth underwent root canal treatment, two teeth were extracted due to periapical lesions and 12 teeth were extracted due to severe periodontitis. The distribution of root canal treatment was upper premolars (*n* = 4, 30.8%), lower molars (*n* = 6, 46.2%), lower premolars (*n* = 1, 7.7%), and lower incisors (*n* = 2, 15.4%). The distribution of extracted teeth was upper molars (*n* = 5, 35.7%), upper premolars (*n* = 2, 14.3%), upper incisors (*n* = 2, 14.3%), lower molars (*n* = 2, 14.3%), lower premolars (*n* = 2, 14.3%), and lower incisors (*n* = 1, 7.1%).

Table 2 presents the relative risk calculations with 95% CI and *p* values between clinical and demographic characteristics and invasive dental treatment after BP. The patients who received invasive dental treatment after BP (*n* = 17, 11.8%) showed a higher comorbidity ACE score of 2–3 (*p* < 0.001), and a higher prevalence of moderate and severe periodontitis (*p* < 0.01), compared with the patients who did not receive invasive dental treatment after BP (*n* = 127, 88.2%).

**Table 2:** Clinical and demographic characteristics and relative risk of dental invasive treatment after BP

	Relative risk (95% CI)
**Gender (male)**	1.53 (0.99–2.06)
**ACE-27 score 2–3**	2.13 (1.74–2.51)***
**Decayed teeth**	1.19 (0.76–1.61)
**Missed teeth**	1.13 (0.44–1.81)
**Periodontitis moderate-severe**	2.33 (1.96–2.67)**
**Apical periodontitis**	1.27 (0.83–1.72)
**No dental treatment prior to BP**	0.79 (0.32–1.25)
**Dental treatment prior to BP**	1.27 (0.81–1.74)

Associations are expressed as RR with 95% CI. Significance levels are presented as * = *p* < 0.05 ** = *p* < 0.01 *** = *p* < 0.001

Among the patients who received invasive dental treatment after BP, no difference was seen in moderate/severe periodontitis occurrence between those with low comorbidity (0–1) and those with high comorbidity [2, 3]; 24% (*n* = 17) and 21% (*n* = 15), respectively.

### Medication related osteonecrosis of the jaw

The incidence rate for development of MRONJ was 2.5/100 person-years, 6.3% (*n* = 9) of the 144 patients included in the study. MRONJ development occurred at a median of 25 months (range 13–50) from the first visit. Of these patients, 77.8% (*n* = 13) were male and 22.2% (*n* = 4) female with a median age of 79 years (range 61–85 years). Their median comorbidity score was 2. All patients who developed MRONJ had received bisphosphonate, chemotherapy, and corticosteroid treatments. The chemotherapy consisted in seven cases of Bortezomib, one case of Bortezomib and Cyclophosphamide, and one case of Bortezomib and Melphalan. However, there was no significant association between monoclonal antibody treatment and the development of MRONJ. On the other hand, the use of three or more treatments as combination antineoplastic therapy showed a strong significance (*p* < 0.001) in the development of MRONJ. Moderate or severe periodontitis was significantly more prevalent in the MRONJ group, with a prevalence of 55.5% (*n* = 5) compared to the group that underwent invasive dental treatment after BP but did not develop MRONJ, with a prevalence of 33.3% (*n* = 4) (*p* < 0.05).

Of those who developed MRONJ (*n* = 9, 6.3%), four patients had tooth extraction (44.4%), and one (11.1%) had a root canal treatment, after the start of BP treatment, at the site of the MRONJ lesion. Six of the nine patients (66.6%) who developed MRONJ had removable prosthetics, but only two patients (22.2%) had removable prosthetics at the site of MRONJ and had undergone tooth extraction. The remaining patients (*n* = 4, 44.4%) developed MRONJ spontaneously, without previous invasive dental treatment or dental infection. The distribution of MRONJ were stage 0 (*n* = 1, 11%), stage 1 (*n* = 5, 56%), stage 2 (*n* = 2, 22%) and stage 3 (*n* = 1, 11%). The most common site to develop MRONJ was the lower molar area (*n* = 5, 55.6%), followed by the upper premolar (*n* = 2, 22.2%), upper molar (*n* = 1, 11.1%) and upper incisor (*n* = 1, 11.1%) areas. The spontaneous MRONJ sites were the lower molar area (*n* = 3, 33.3%) and the upper premolar area (*n* = 1, 11.1%). If the patient had ongoing BP treatment at the time of MRONJ diagnosis, this was terminated. Six patients (66.6%), (MRONJ stages 0–3) received non-operative treatment and antibiotics if signs of infection which led to sequestration and healing in two cases (33.3%). Three patients (33.3%), (MRONJ stages 1–2) received combination of non-operative therapy and operative therapy by surgical resection which led to healing in two cases (66.6%).

The incidence of development of MRONJ in patients who did not undergo invasive dental treatment after BP was significantly lower (*n* = 4, 3.2%) compared with those who did (*n* = 5, 20%) (*p* < 0.001). The attributable risk factor of invasive dental treatment in the development of MRONJ was 4.47.

Table 3 presents the relative risk calculations with 95% CI and level of significance between clinical and demographic characteristics and MRONJ. When comparing patients who developed MRONJ (*n* = 9, 6.3%) and those who did not develop MRONJ (*n* = 135, 86.7%), a larger number of missed teeth, a higher prevalence of moderate and severe periodontitis, removable prosthetics, 3 or more treatment combinations and invasive dental treatment after BP were significant risk factors associated with the MRONJ incidence.

**Table 3:** Clinical and demographic characteristics and risk of Medication-Related Osteonecrosis of the Jaw

	Relative risk (95% CI)
**Gender** (male)	1.75 (0.97–2.53)
**ACE-27 score 2–3**	2.00 (1.31–2.69)
**Decayed teeth**	2.23 (1.59–2.87)
**Missed teeth**	2.87 (1.83–3.91)**
**Periodontitis moderate-severe**	4.21 (3.57–4.84)***
**Apical periodontitis**	0.27 (−0.78–1.32)
**Removable prosthetics**	3.70 (2.82–4.58)*
**Dental treatment prior to BP**	0.79 (0.10–1.47)
**Dental treatment after BP**	7.75 (7.14–8.36)***
**Monoclonal antibodies treatment**	1.57 (0.89–2.25)
**3 or more treatment combinations**	4.13 (3.45–4.81)**

Associations are expressed as RR with 95% CI. Significance levels are presented as  * = *p* < 0. 05 ** = *p* < 0.01 *** = *p* < 0.001

### Osteolytic lesions

The prevalence of general osteopenia or osteolytic lesions in the jawbones was 42.3% (*n* = 61) and associated with an increased relative risk of death by 1.8 (95% CI 1.38–2.27, *p* < 0.05). The maxilla was only involved in 15.6% (*n* = 9) of these cases, while the mandible showed lesions in all cases. Osteopenia was seen in 20% (*n* = 12) and osteolytic lesions in 80% (*n* = 49), which presented as solitary (*n* = 11, 22.2%) or scattered (*n* = 38, 77.5%) lesions. The sites of osteolytic lesions within the mandible were the ramus (*n* = 33, 68.9%), corpus (*n* = 14, 28.9%), and processus alveolaris (*n* = 12, 24.4%). Symptoms from the lesions (loss of sensation, tooth mobility and soft tissue swellings) developed in 13.3% (*n* = 6) of the cases and 11.1% (*n* = 5) received external radiation therapy or intensified treatment with corticosteroids and chemotherapy. No patient developed a pathological fracture of the mandible.

### Oral health related quality of life

**Fig. 2:**
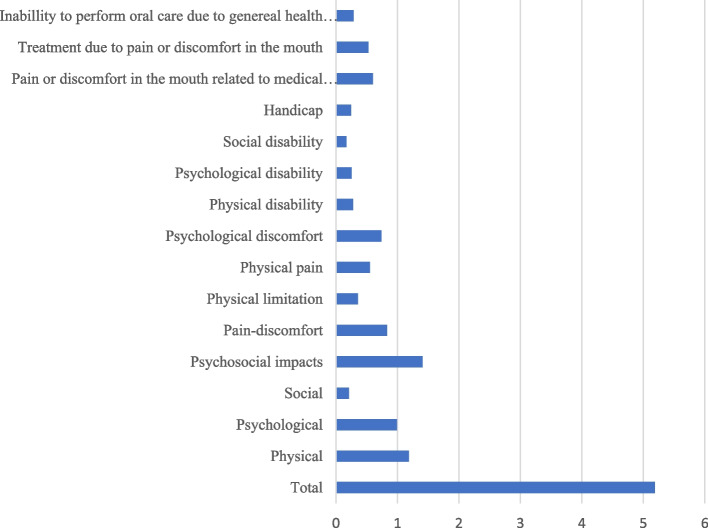
Mean oral health impact profile 14 (OHIP-14) score in the study sample (*n* = 59)

The response rate of the OHIP-14 questionnaire, including the three additional questions, was 70.2% (*n* = 59). Among the responders, 64.5% (*n* = 38) were male and 35.5% (*n* = 21) were female. The median age 74 (49–85) and median comorbidity ACE − 27 score 2 (0–3). A summary of the responses can be found in Fig. 2. The results are presented in a third-order hierarchal model, a second-order hierarchical model with three first-order factors and the seven first order factors. Psychological discomfort followed by physical pain were the problems most often reported by the patients. Oral pain or discomfort in the mouth related to the medical treatment was reported by 30.5% (*n* = 18) of the patients. Of those who reported oral pain or discomfort, 72% (*n* = 13) also reported that they had received treatment for pain in the oral cavity. According to the results of the OHIP − 14 questionnaire, only 18.6% (*n* = 11) of the patients had experienced an inability to perform daily oral hygiene routines due to their impaired health status.

## Discussion

As far as we know, this is the first longitudinal cohort study where all patients diagnosed with multiple myeloma in a healthcare area were followed regarding oral health status and oral health-related quality of life. The results of this study showed that all patients who developed MRONJ had received BP, chemotherapy, and corticosteroid treatments, and emphasizes invasive dental treatment (tooth extraction and root canal treatment) as the greatest risk factor for MRONJ in MM patients undergoing BP. Moreover, periodontitis, three or more treatment combinations, removable prosthetics, and a larger number of missed teeth were also associated with MRONJ development. However, increasing age and greater comorbidity did not imply a significantly increased risk of developing MRONJ.

According to the 2022 position paper by the AAOMS, increased comorbidity and age are inconsistently reported as risk factors in the development of MRONJ. Concomitant treatment with corticosteroids and longer duration of BP treatment are associated with an increased risk of MRONJ [7]. The Cochrane review from 2017 states that the incidence of MRONJ in cancer patients, including MM patients, exposed to IV zoledronic or pamidronic acid ranges from 0.3–5% [27]. A review by Rugani et al. [28] comprising 69 reports on the prevalence of MRONJ in different malignancies found that the highest prevalence of MRONJ in MM was 5.2%, followed by prostate cancer (3.8%), and breast cancer (2.1%). Our study showed a slightly higher incidence of MRONJ, 6.3%, which may be explained by frequent clinical follow-ups during the study, allowing for an early diagnosis of MRONJ. It is noteworthy that 3.2% of the patients developed MRONJ without evidence of trauma, invasive dental treatment or infection, while 20% developed MRONJ after invasive treatment. This emphasises the importance of regular and careful dental follow-up to minimise the need for invasive treatment in the future.

In Sweden, all patients with malignant haematological diseases and/or in need of treatment with high-dose BPs are referred to a specialist dental clinic for orofacial medicine for a dental examination and treatment of oral infections. Clinical dental care guidelines during HSCT are available, but no guidelines exist regarding dental care prior to BP treatment and appropriate follow-up. The long half-life of BP presents the dentist with the difficulty not only to remove existing dental infections, but also to prevent the occurrence of dental infections in the future, as infection and tooth extraction are the most frequent aetiological factors for the development of MRONJ [29, 30].

Among the MM patients in the present study, 42% showed general osteopenia and/or osteolytic lesions in the jawbones as signs of MM manifestation, which is slightly higher than in a previous study [13]. In the present study, MM patients with manifestation in the jaw bones had a poorer disease prognosis, which is in line with a previous study [14]. The development of osteolytic lesions in MM is also an important aspect for the dental practitioner, as it may lead to loss of sensation, tooth mobility, soft tissue swellings, and mandibular fractures. As a supplement to computed tomography, panoramic radiographs are effective to detect myeloma-related osteolytic lesions and determine their size [31].

The median DMFT score presented in this study was 21, which is in line with the results from the latest oral health survey of persons in the same age groups in Jönköping, Sweden, performed in 2013 [32], and with a European study showing a median DMFT of 22 [33]. Another study showed no differences in the number of decayed, missed or filled teeth between patients with MM and healthy controls [15].

The periodontal status of MM patients in this study corresponds well to that of persons in the same age group included in the study by Norderyd et al., 2015, and to a review of the epidemiological data on periodontal disease in Europe [34].

In the present study, 33% of the patients had periapical lesions, which is in accordance with a previous study by Oñate-Sánchez et al., 2020. There was no difference in the presence of periapical lesions in MM patients compared with healthy controls [35].

Taken together, the results of our study on the oral health status of MM patients are in accordance with previous studies [15, 32–35], showing that individuals diagnosed with MM have an oral health status comparable to that of an age-matched normal population prior to the start of BP therapy.

A plausible explanation is that Sweden has a tradition of routine dental care, where 80% of the adult population receive regular dental care and 52% visit the dental service annually [36]. Hence, the follow-up visits in the present study are no different from what most of the patients would receive in the form of regular dental care, even if they had not been diagnosed with myeloma. In Sweden, dental examinations and treatment in myeloma patients are included in the health care system and all newly diagnosed patients are referred to a specialist clinic for orofacial medicine. As the patients pay a small fee for the visit, instead of the ordinary dental fee, socioeconomic vulnerability does not affect their access to dental care. Thus, a strength of present study is that there is no selection bias due to economy or accessibility.

The OHRQoL in MM patients during HSCT in relation to the severity of the oral mucositis was investigated by Periera et al., 2018. It was found that there was little influence on OHRQoL when no mucositis was present. As the mucositis rate increased, a decrease in OHRQoL was seen [37]. No previous study has investigated the OHRQoL of a cross-section of myeloma patients as the majority are not eligible for HSCT due to age over 70 and high comorbidity.

According to the results of the OHIP-14 questionnaire, only 18.6% of the patients had experienced an inability to perform daily oral hygiene routines due to their impaired health status. This may indicate that although MM patients suffer from an incurable disease, their motivation and physical ability to perform daily oral hygiene are rarely affected. Oral pain or discomfort in the mouth related to the medical treatment was reported by 30.5% of the patients. It is well known that MM patients may have oral pain and discomfort due to oncological treatments, including HSCT and chemotherapy, and later due to MRONJ development as an adverse effect of BP treatment. However, there are no cross-sectional studies that have focused on oral health-related problems in MM patients. Our results demonstrate a large variation in OHRQoL where the majority experience good OHRQoL, but some individuals experience a great negative impact, both physically and psychologically. Further research is needed to investigate the vast differences in OHRQoL and the possibility of improvement.

A limitation of the study is that no bone biopsy was performed of the alveolar bone during tooth extraction to exclude the possibility that the patient had already developed MRONJ at the site prior extraction which has been shown in patients with symptomatic teeth receiving bone-targeting agents [38]. Unfortunately, in Sweden, there is no routine for alveolar bone biopsy in connection to the dental extraction why this has not been investigated. Furthermore, it is a weakness that it is a retrospective study and 13 different clinicians have been involved in the dental examination and treatment of the patients, which may cause inter-examiner variation. However, weekly sessions between the clinicians discussing findings from the clinical and radiological examination and treatment options were held over the years. Also, close collaboration with other odontological specialities and the haematology department was maintained throughout the study. The study was not blinded since the principal investigator RO performed the data collection.

## Conclusions

The knowledge gained from the study may act as an aid for dental clinicians in the treatment planning and screening of patients with an increased risk of invasive dental treatment and MRONJ. This would result in fewer oral infections and fewer interruptions of the antineoplastic treatment, which benefits general health, OHRQoL and health economics. The results of this study may also make general dental practitioners more aware of the risk of MRONJ development in MM patients due to invasive dental treatments and the high occurrence of osteolytic lesions, mainly in the mandible.

## Data Availability

The dataset generated and analysed during the current study is available from the corresponding author on reasonable request.
